# Whole Mount *in situ* Localization of miRNAs and mRNAs During Somatic Embryogenesis in Arabidopsis

**DOI:** 10.3389/fpls.2018.01277

**Published:** 2018-09-04

**Authors:** Anna M. Wójcik, Magdalena Mosiolek, Jagna Karcz, Michael D. Nodine, Małgorzata D. Gaj

**Affiliations:** ^1^Department of Genetics, Faculty of Biology and Environmental Protection, University of Silesia, Katowice, Poland; ^2^Gregor Mendel Institute of Molecular Plant Biology, Austrian Academy of Sciences, Vienna, Austria; ^3^Scanning Electron Microscopy Laboratory, Faculty of Biology and Environmental Protection, University of Silesia, Katowice, Poland

**Keywords:** WISH, miRNA, mRNA, somatic embryogenesis, Arabidopsis, *in situ* hybridisation, *in vitro*

## Abstract

Somatic embryogenesis (SE) results from the transition of differentiated plant somatic cells into embryogenic cells that requires the extensive reprogramming of the somatic cell transcriptome. Commonly, the SE-involved genes are identified by analyzing the heterogeneous population of explant cells and thus, it is necessary to validate the expression of the candidate genes in the cells that are competent for embryogenic transition. Here, we optimized and implemented the whole mount *in situ* hybridization (WISH) method (Bleckmann and Dresselhaus, 2016; Dastidar et al., 2016) in order to analyze the spatiotemporal localization of miRNAs (miR156, miR166, miR390, miR167) and mRNAs such as *WOX5* and *PHABULOSA*-target of miR165/166 during the SE that is induced in Arabidopsis explants. This study presents a detailed step-by-step description of the WISH procedure in which DIG-labeled LNA and RNA probes were used to detect miRNAs and mRNAs, respectively. The usefulness of the WISH in the functional analysis of the SE-involved regulatory pathways is demonstrated and the advantages of this method are highlighted: (i) the ability to analyze intact non-sectioned plant tissue; (ii) the specificity of transcript detection; (iii) the detection of miRNA; and (iv) a semi-quantitative assessment of the RNA abundance.

## Introduction

Somatic embryogenesis (SE) involves the formation of somatic embryos in somatic cells that are cultured *in vitro*, and therefore, it provides a model system to study the developmental plasticity of plant somatic cells. Because SE results in the efficient production of somatic embryo-derived regenerates, it has become a powerful tool in plant biotechnology, and is widely applied in the clonal propagation and genetic transformation of numerous plant species (reviewed Misra and Saema, 2016). Moreover, considering the similarities between SE and zygotic embryogenesis (ZE), the functional genomics of SE could facilitate the analysis of the molecular mechanisms of ZE (Zimmerman, 1993). Various molecular tools have been employed to identify the genes that are essential for the embryogenic transition of somatic plant cells and several genes that have a regulatory function, mainly encoding transcription factors, were discovered in the last decade. In contrast, data on the involvement of miRNA in SE is limited primarily because of limitations in the availability of the research tools that are required for such analyses. Following reports on a key role of miRNAs in ZE in Arabidopsis (Nodine and Bartel, 2010; Seefried et al., 2014; Armenta-Medina et al., 2017), the central function of *MIRNA* genes in embryogenic transition *in vitro* can be assumed. In support of the hypothesis of the involvement of miRNA in SE, mutations in *DICER-LIKE1* (*DCL1*), which is required for miRNA biogenesis, have been found to inhibit SE-induction *in vitro* in cultures of Arabidopsis explants (Wójcik and Gaj, 2016). The hypothesis has also been supported by the analysis of more than 190 *MIRNA* in Arabidopsis, which showed that more than 20 genes were differentially regulated during SE induction (Szyrajew et al., 2017). Similar to Arabidopsis, the differential expression of miRNAs was reported in embryogenic cultures of other plants, including *Oryza sativa* (Chen et al., 2011), hybrid yellow poplar (Li et al., 2012), *Larix laptolerix* (Zhang et al., 2012), *Dimocarpus longan* (Lin and Lai, 2013), *Gossypium hirsutum* (Yang et al., 2013), and *Zea mays* (Chávez-Hernández et al., 2015).

The current limitation for identifying SE-enriched genes/miRNA is to distinguish the cells that are triggered toward embryogenic development from the surrounding explant cells. One way to examine whether the expression of the analyzed gene is present in cells that are undergoing SE is to analyze the reporter lines, which are based on the GUS or GFP constructs. However, the overwhelming majority of the available reporter lines monitor promoter activity rather than lead to the localization of the corresponding transcript. In order to overcome the time-consuming and laborious processes that are required to prepare a specific construct and plant transformation, we present the whole mount *in situ* hybridization (WISH) protocol for miRNA and mRNA molecules in explants that are undergoing SE induction.

## Materials and Methods

### Plant Material and Growth Conditions

The *Arabidopsis thaliana* (L.) Heynh. Columbia (Col-0) parental genotype and the transgenic lines *35S::MIR156* (N9952) and *35S::MIM156* (N9953) were supplied by the Nottingham Arabidopsis Stock Centre (NASC). The seeds of the *2x35S::STTM165/166* line (hereafter referred to as STTM165/166) were kindly provided by Dr. Guiliang Tang (Michigan Technological University, United States), while the seeds of the *P_MIR167a_::GUS* and *P_MIR167c_::GUS* lines were kindly provided by Jason W. Reed from the Department of Biology, University of North Carolina at Chapel Hill.

The seeds were sterilized using a 20% solution of commercial bleach and then plated on a solid 1/2 MS medium. The plates were kept in a growth chamber at 21 ± 1°C under a 16/8 h photoperiod of 40 μM m^-2^s^-1^ white, fluorescent light. The young seedlings were transferred into Jiffy-7 pots and grown in a walk-in type green room under the conditions that are described above until the siliques were harvested.

### Somatic Embryogenesis Induction

Immature zygotic embryos (IZEs) at the green cotyledonary stage were used as the explants to induce SE. The IZEs were cultured in an E5 solid medium with 5 μM 2,4-dichlorophenoxyacetic acid (2,4-D) according to Gaj (2001) for 5, 10, 15, and 21 days.

### Scanning Electron Microscopy (SEM)

Explants from different time points of the SE culture were collected and fixed in 4% PFA in PBS with 0.1% Tween in a vacuum for 3 h at room temperature (RT). Next, the samples were rinsed for 30 min in methanol and then washed 3 × 5 min in 100% ethanol. After fixation, the samples were washed in PBS and dehydrated in an ethanol series (30, 50, 70, 80, 90, 95, and 100%) for 10 min each, followed by replacing the ethanol with acetone. The dehydrated samples were dried with a CPD 2 critical-point drier (Pelco) using liquid carbon dioxide, mounted on aluminum stubs with double-sided adhesive carbon tape and sputter-coated with a 12.5 nm (0, 5, 10, 15 days) or 20 nm (21 and 28 days) film of gold in an SC-6 sputter coater (Pelco). After processing, the samples were imaged using a Hitachi SU 8010 UHR FESEM field emission scanning electron microscope (Hitachi High-Technologies Corporation, Tokyo, Japan) at a 5 kV accelerating voltage with a secondary electron detector (ESD).

### Light Microscopy (LM)

After stopping the colorimetric detection, the explants were transferred to three-welled glass slides, mounted in 70% glycerol in a TE buffer or Clear Solution and sealed with cover-slips. The slides were subsequently imaged on an automated Pannoramic SCAN 150 slide scanner (3D HISTECH) with transmitted light and a 20X plan-apochromat objective or with an Olympus BX-43 microscope. Images of the embryos were collected using the Panoramic Viewer software (3DHISTECH) or with an Olympus SC-30 camera using the CellSens software.

### Probe Design

For the miRNA WISH procedure, LNA-modified oligonucleotide probes that corresponded to the full antisense sequence of the miRNA were DIG-labeled at both the 5′ and 3′ ends that contained 4 LNA-modified bases at positions 7, 9, 11, 15. The probes were synthesized using Exiqon.

The probes that were used for the mRNA WISH were synthesized as ∼450 bp cDNA transcripts, and then cloned into pSPT19. The T7/SP6 RNA polymerases were used to generate DIG-labeled probes by an *in vitro* transcription reaction using an NTP Labeling Mix.

### Reagents

• Plant material: immature zygotic embryos that were cultured on an SE induction medium for 0, 5, 10, 15 days); 60 explants per culture combination
• Formamide (Merck, F9037)
• NBT/BCIP Stock solution (Roche, 11681451001)
• Anti-Digoxigenin-AP (Roche, 11093274910)
• 1-Methylimidazole (Merck, 336092)
• N-(3-Dimethylaminopropyl)-N′-ethylcarbodiimide hydrochloride (Merck, E7750)
• BSA solution 30% (Merck, A8577)
• Denhardt’s solution (Merck, 30915)
• Dextran sulfate 50% solution (Merckmillipore, S4030)
• tRNA 50 mg/ml (Roche, 10109541001)
• Histo-Clear II (Electron Microscopy Science, 64111-01)
• SSC buffer 20 × concentrate (Merck, S6639)
• Glycine (Merck, 410225)
• Tween-20 (Merck, 27.434-8)
• Proteinase K (Thermo Fisher Scientific, EO0491)
• Sodium acetate (Merck, cat. no. 6268.1000)
• T7 RNA polymerase (Roche, 10881767001)
• SP6 RNA polymerase (Roche, 10810274001)
• NTP Labeling Mix (Roche, II277073910)
• Methanol (POCH, 621990110)
• Ethanol (POCH, 396480111)
• Formaldehyde solution 37% (Merck, 252549)
• RNaseZAP (Merc, R2020)

### Equipment

• Medium incubation baskets (100 μm) (Intavis, 12.440)
• 24-well cell culture clusters (Costar, 3526)
• “PTFE” Slides (Electron Microscopy Science, 63418-11)
• ExPellPlus Filter Tip (Biokom, 5130150, 5030090, 5030030)
• Glass bottles, sterile, nucleases free (baked 8h at 180°C)
• RNase-free Microfuge Tubes (1.5 mL) (Thermo Fisher Scientific, AM12400)
• Cover slips 24 mm × 50 mm (Menzel-Glaser)
• Tweezers

### Buffers

• Tris–HCl 1 M [250 ml]: 30.275 g Tris + up to 250 ml H_2_O; add pure HCl till pH 7.5; 8.0 or 9.0
• EDTA 0.5 M [250 ml]: 46.525 g EDTA + up to 250 ml H_2_O; add crystals of NaOH (∼0.5 g) till pH 8
• NaCl 5 M [250 ml]: 73.05 g NaCl + up to 250 ml H_2_O
• MgCl_2_ 1 M [100 ml]: 20.3 g MgCl_2_ × 6H_2_O + up to 100 ml H_2_O
• RNase buffer 5× [500 ml]: 25 ml 1 M Tris–HCl pH 7.5 [50 mM] + 5 ml 0.5 M EDTA [5 mM] + 250 ml 5 M NaCl [2,5 M] + up to 500 ml H_2_O; pH 7.5
• Clearing solution [1 ml]: 2.5 g chloral hydrate in 1 ml 30% glycerol in H_2_O
• 2× carbonate buffer [100 ml]: 1.277 g Na_2_CO_3_ [120 mM] + 0.672 g NaHCO_3_ [80 mM] + up to 100 ml H_2_0 – DEPC; pH 10.2 [NaOH]
• PBS 10× [1000 ml]: 76 g NaCl + 4.14 g NaH_2_PO_4_ + 25.07 g Na_2_HPO_4__+_ up to 1000 ml H_2_O; pH 7.0 [HCl]
• Proteinase K buffer [200 ml]: 100 ml 1 M Tris pH 7.5 + 100 ml 0.5 M EDTA + 0.5 ml acetic anhydride (DO NOT TREAT WITH DEPC!)
• PBST: PBS + 0.1% Tween (1 μl of Tween per 1 ml PBS)
• Na_2_HPO_4_ 1 M [1000 ml]: 141.96 g Na_2_HPO_4_ + up to 1000 ml H_2_O
• NaH_2_PO_4_ 1 M [1000 ml]: 138 g NaH_2_PO_4_ + up to 1000 ml H_2_O
• Na-phosphate [100 ml]: 46.3 ml Na_2_HPO_4_ 1 M + 53.7 ml NaH_2_PO_4_ 1 M; pH 6.8
• *In situ* salts 10× [10 ml]: 6 ml 5 M NaCl + 1 ml 1 M Tris pH 8.0 + 1 ml 1 M Na-phosphate + 1 ml 0.5 M EDTA + 1 ml H_2_0
• Hybridization Mix [10 ml]: 5 ml formamide + 2 ml 50% dextran sulfate + 1 ml 10× *in situ* salts + 0.2 ml 50× Denhardt’s salts + 0.1 ml tRNA 50 mg/ml + 1.7 ml H_2_O-DEPC (fresh)
• TNM-50 [1000 ml]: 100 ml 1 M Tris pH 9.5 + 20 ml 5 M NaCl + 50 ml 1 M MgCl_2_ + 830 ml H_2_O-DEPC (fresh)
• TE 10× [1000 ml]: 100 ml 1 M Tris pH 8.0 + 20 ml 0.5 M EDTA + up to 1000 ml H_2_O
• Methylimidazole-NaCl [1000 ml]: 10.36 ml methylimidazole + 75 ml NaCl 5 M + up to 1000 ml H_2_O; pH 8.0/HCl (pH is very important!)
• EDC [1000 ml]: 31.632 g N-(3-Dimethylaminopropyl)-N′-ethylcarbodiimide hydrochloride + up to 1000 ml methylimidazole-NaCl pH 8.0

### Stepwise Procedures

#### WISH of the miRNA Molecules

The available protocol of WISH (Dastidar et al., 2016) was modified and optimized for explants undergoing SE induction. Accordingly, each step of the procedure was optimized in terms of duration of treatments, amount of washings, composition of buffers and probe/enzymes concentration. During the procedure to prepare the liquid solutions, RNase-free water and RNase-free baked glass bottles should be used. Whenever possible, the buffers should be treated with DEPC. The full list of buffers and solutions can be found in the section “Materials and Methods.” To facilitate frequent buffer changes, the entire procedure can be carried out in medium incubation baskets (100 μm) (Intavis), washed in 24-well plates with lids. The tissue should be covered with the buffers in the wells (from 0.6 ml to 1.0 ml of the buffer/reagent per well). Until the hybridization step is finished, only RNase- and DNase-free barrier pipette tips should be used.

### Procedure

#### Tissue Collection and Fixation (Timing 3.5 h+)

1. Depending on the age of the explants in the culture, different approaches were developed. Freshly isolated explants from the siliques (0 day of culture) were dissected in a drop of PBS, while the explants after 5, 10, and 15 days of SE culture were transferred directly from the medium onto 3.7% PFA in PBST (PBS + 0.1% of Tween^®^ 20) on ice. The tissue was fixed under a vacuum for 3 h at RT.

⊕ PAUSE: The fixation may also proceed overnight at 4°C without vacuum.

! NOTE: The incubation baskets should be washed with RNAseZAP and then in H_2_O-DNase and RNase free. Put the baskets into the wells in the culture cluster using tweezers and fill it with 3.7% PFA in PBST.

! NOTE: The recommended fixation for 10 days or older tissues is 3 h under a vacuum at RT and overnight at 4°C without vacuum.

#### Dehydration (Timing 20 min – A Few Weeks)

2. Incubate the tissue in 100% methanol until the chlorophyll is removed (at least three times for 5 min), then twice in 100% ethanol for 5 min each at RT.

! NOTE: For 10 days and older tissues, the incubation in methanol can take up to 1 h at RT.

⊕ PAUSE: The fixed and dehydrated tissue can be stored in 100% ethanol at -20°C for several weeks.

#### Permeabilization (Timing 5 h)

3. Incubate for 30 min in 1:1 Histo-Clear II and a 100% ethanol solution at RT.
4. Wash twice for 5 min in 100% ethanol at RT.
5. Rehydrate in 90, 70, 50, and 30% ethanol/H_2_O, 10 min each at RT.
6. Incubate in a proteinase K buffer for 5 min at RT.
7. Incubate with proteinase K (75 μg/ml) in a proteinase K buffer for 15 min at 37°C.

! NOTE: The proteinase K buffer should be preheated to 37°C.

8. Stop the digestion using glycine (2 mg/ml) in PBS by incubating it for 5 min at RT.
9. Wash for 5 min in PBST at RT.
10. Refix in 3.7% PFA in PBST for 10 min at RT.
11. Wash twice in PBST for 5 min each at RT.
12. Incubate three times for 10 min each in methylimidazole-NaCl (pH 8.0) at RT.
13. Incubate for 2 h in 0.16 M EDC in methylimidazole-NaCl at 60°C.
14. Wash twice for 10 min in PBST at RT.

#### Probe Hybridization (Timing 12 h/Overnight)

15. Wash in a pre-hybridization mix (hybridization mix without the probe) for 10 min at RT.
16. Denature the probe in the hybridization mix for 2 min at 80°C.
17. Incubate 12 h or overnight at 65°C in the hybridization mix with the denatured probe.

! NOTE: The hybridization mix should be prepared fresh shortly before use.

! NOTE: The best probe concentration was 20 nM.

! NOTE: The incubation at 65°C may be carried out in a water bath in a 24-well plate that is sealed by Parafilm or in a humidified chamber.

#### Washing (Timing 1.5 h)

18. Wash twice for 10 min in 2×SSC at RT.
19. Incubate twice for 30 min in 0.2×SSC at 65°C.

! NOTE: During the incubation in 0.2×SSC at 65°C, the probes should be shaken from time to time OR the incubation can be carried out on a shaker at 65°C.

20. Wash 5 min in PBST at RT.

#### Antibody Binding (Timing 3 h)

21. Incubate in 1% BSA in PBST for 30 min on a shaker at RT.
22. Incubate with Anti-Digoxigenin-AP (1:1250) in 1% BSA in PBST for 90 min on a shaker at RT.
23. Incubate in 1% BSA in PBST for 30 min on a shaker at RT.
24. Wash twice for 10 min in PBST at RT.

#### Probe Detection (Timing 1 h+)

25. Wash three times in TNM-50, 5 min each at RT.
26. Incubate in NBT-BCIP (1:50) in TNM-50 at RT.

! NOTE: The TNM-50 buffer and NBT-BCIP solution should be freshly prepared, shortly before use.

! NOTE: The incubation in NBT-BCIP can be carried out at RT or at 37°C in the dark. If the incubation is at 37°C, the colorimetric reaction should be checked every 10 min.

! NOTE: During the incubation at RT colorimetric reaction for miR390 takes 1 h, while takes at least 3 h for miR124 and miR166.

27. Stop the reaction using TE and wash three times for 5 min in TE at RT.

#### Mounting and Microscope Detection

28. Carefully transfer the explants in TE onto glass slides.
29. Remove the TE and add the 70% glycerol in TE or clearing solution, gently cover with a cover slip.
30. Perform the microscope analysis.

! NOTE: Do not allow the explants to remain in the clearing solution for longer than 30 min.

#### WISH of the mRNA Molecules

To perform the mRNA WISH in explants that are undergoing the SE process, we modified and adjusted the procedure based on alkaline phosphatase (AP) coupled antibody detecting the anti-Digoxigenin labeled probes using BCIP-NBT substrates (Bleckmann and Dresselhaus, 2016). The tips, notes and procedure are quite similar to those for miRNA WISH that are presented above with a few changes that are itemized below.

### Procedure

1. - 2. Proceed with *tissue collection, fixation, and dehydratation* as described in the procedure for the miRNA WISH.
3 - 11. The *Permeabilization* steps are performed as in the miRNA WISH procedure but omitting steps 12–14.

! NOTE: Although the EDC cross-links the small RNA 5′ ends to the protein matrix, which has a superior sensitivity for miRNA-WISH, it is no longer needed in the RNA probes in the mRNA-WISH.

12. Perform the *hybridization* as in the miRNA WISH with a hybridization temperature of 55°C and a probe concentration of 0.5 ng/μl.

#### Washing and RNase A Treatment (Timing 3 h)

13. Wash the probes in 50% formamide in 2×SSC + 0.1% Tween once for 10 min and once for 30 min at 55°C.
14. Wash in 2×SSC twice for 5 min at RT.
15. Wash in 0.2×SSC for 30 min at 55°C.

! NOTE: During the incubation in 0.2×SSC, the probes should be shaken from time to time OR the incubation can be carried out on a shaker at 55°C.

16. Wash twice for 10 min in the RNase buffer at RT.
17. Incubate 15 min in RNase A (25 μg/ml in RNase buffer) at 37°C.
18. Wash three times for 5 min in the RNase buffer at RT.

! NOTE: The RNase buffer should be preheated to 37°C before use.

19. Wash in 0.2×SSC for 30 min at 55°C.

! NOTE: During the incubation in 0.2×SSC, the probes should be shaken from time to time OR the incubation can be carried out on a shaker at 55°C.

20. Wash twice for 10 min in PBST.

#### Antibody Binding (Timing 4 h+)

21. Incubate in 1% BSA in PBST for 90 min on a shaker at RT.
22. Incubate with Anti-Digoxigenin-AP (1:1500) in 1% BSA in PBST for 4 h on a shaker at RT.

⊕ PAUSE: The incubation with the antibody can proceed overnight at RT.

23. Incubate in 1% BSA in PBST for 60 min on a shaker at RT.
24. Wash three times for 10 min in PBST at RT.

*Probe detection, mounting, and microscope detection* should be performed as was described in steps 25–30 for the miRNA-WISH procedure.

## Results and Discussion

**FIGURE 1:**
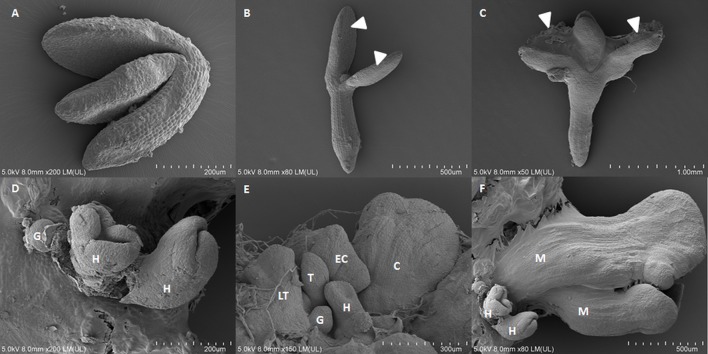
Scanning electron microscopy images of the somatic embryogenesis culture of Arabidopsis from immature zygotic embryos on a medium with 5 μM of 2,4-D on 0th day **(A)**, 5th day **(B)**, 10th day **(C)**, and 15th day **(D–F)** of the culture. **(D–F)** Somatic embryos in the globular (G), heart (H), torpedo (T), late torpedo (LT), early cotyledonary (EC), cotyledonary (C), mature stages of development (M), arrow heads indicate tissue that is undergoing SE.

Identifying the SE-specific miRNA molecules and genes is crucial to understanding the mechanisms that govern the developmental plasticity of plant cells. Different approaches have been used to determine whether the candidate transcripts that have been selected by expression analysis are present in the cells that are undergoing SE induction within the mass of the explants’ cells. Analyzing SE induction is difficult because of the heterogeneous population of explant cells in which only a small fraction is capable of responding to embryogenic induction. It has been demonstrated that SE induction occurs in the upper part of the explants, preferentially on the adaxial side of the cotyledons. Moreover, somatic embryos can be of a single-cell or multicellular origin and that they develop asynchronously from both the protodermal and subprotodermal cell layers (Kurczyńska et al., 2007; Singh et al., 2015; **Figure 1**). Because of such heterogeneous cell populations, the results from RT-qPCR on whole explants are often difficult to interpret. Therefore, identifying the SE-associated genes requires insight into spatiotemporal expression patterns of the candidate genes. Hence, based on the original protocols for the direct localization of miRNA and mRNA using *in situ* hybridization with specific anti-sense LNA (locked nucleic acid) probes for miRNA molecules (Bleckmann and Dresselhaus, 2016; Dastidar et al., 2016), we modified and optimized the procedure for explant tissue that is undergoing SE.

**FIGURE 2:**
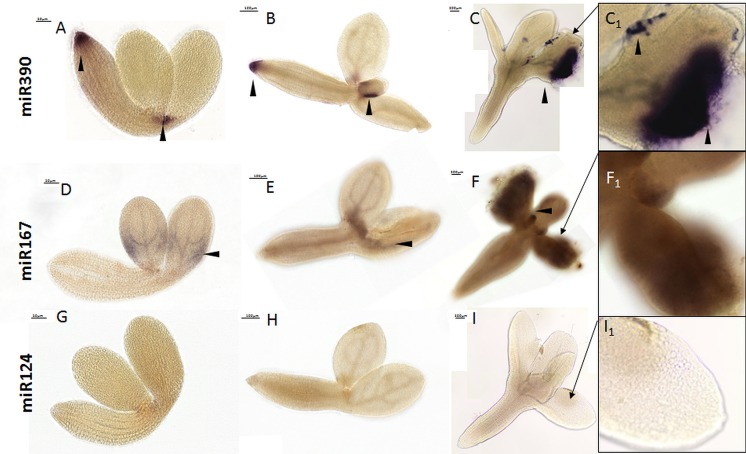
Whole mount *in situ* hybridization of the miR390 and miR167 molecules in the explants undergoing SE induction in 0th day **(A,D,G)**, 5th day **(B,E,H)**, and 10th day **(C,F,I)**. Probe against the mouse miR124 were used as the negative control. A higher magnification views of the SE-involved cotyledons on the 10th day of the SE culture are presented in black boxes (C_1_, F_1_, and I_1_). Arrow heads indicate the detected signal.

As a positive control, we performed WISH for miR390, which has been found to be expressed in the shoot and root meristem region in zygotic embryos and seedlings (Marin et al., 2010; Wong et al., 2011; Dastidar et al., 2016; Hobecker et al., 2017). Consistent with these reports, the zygotic embryo explants (0 day) displayed the miR390 signal in the root and shoot meristems. The explants that were cultured for 5 days on the SE induction medium also had a similar pattern of miR390 expression (**Figures 2A,B**), while on the 10th day, the WISH signal was localized exclusively in the cotyledons that were engaged in the somatic embryo formation (**Figure 2C**). Given that miR390 controls the lateral root development (Marin et al., 2010), the accumulation of miR390 in the SE-involved cotyledons supports the postulated similarities between the regulatory pathways that control the lateral root induction and *in vitro* plant regeneration, including SE induction (Sugimoto et al., 2011; Fehér et al., 2016).

A signal for miR167 was barely detectable in the cotyledons (**Figure 2D**) and the proximity of SAM in SE-induced explants (**Figures 2E,F**). The weak signal of miR167 corresponded to the results of the RT-qPCR analysis that showed a decreasing level of mature miR167 during SE culture (**Supplementary Figure S1**). Similarly, a low level of miR167 during the induction stage of SE was observed in Longan (Lin and Lai, 2013), the Valencia sweet orange (Wu et al., 2011) as well as in an embryogenic culture of Arabidopsis, where miR167 was suggested to negatively control SE induction by targeting the *ARF6* and *ARF8* genes (Su et al., 2015b). The results of the performed WISH were verified by analyzing the mouse-specific miR124 as a negative control (**Figures 2G–I**).

**FIGURE 3:**
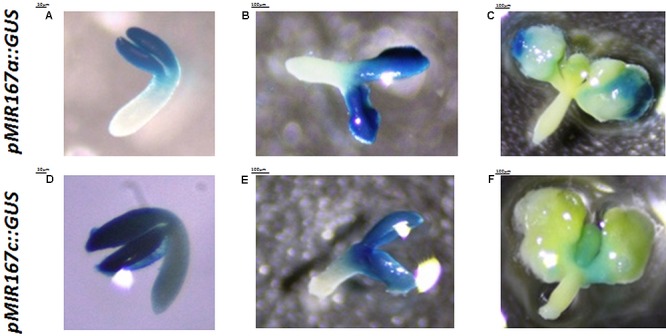
Patterns of the activity of the GUS-monitored promoters of the *MIR167a* **(A–C)** and *MIR167c* **(D–F)** genes at 0th day **(A,D)**, 5th day **(B,E)**, and 10th day **(C,F)** of the SE culture.

The reporter lines that have promoter fusions are the most common tools for the spatiotemporal analysis of the genes that are involved in SE induction (Nolan et al., 2009; Bai et al., 2013; Mozgová et al., 2017; Wójcikowska and Gaj, 2017), including the *MIRNA* genes (Su et al., 2015b; Wójcik and Gaj, 2016). However, a reporter line analysis might not reflect the localization of the functional products of *MIRNA*s due to the extensive post-transcriptional regulation of miRNA biogenesis during plant and animal development (Lee et al., 2008; Nogueira et al., 2009; Bielewicz et al., 2013; Barciszewska-Pacak et al., 2015). Accordingly, the cotyledons and proximity of SAM showed an intense GUS signal of *MIR167a* and *MIR167c* promoter activity during all of the stages of SE induction that were analyzed (**Figure 3**), which did not correspond to the low amount of mature miR167 molecules that were found in the SE-induced explants using the RT-qPCR (**Supplementary Figure S1**) and WISH (**Figures 2D–F**) method.

**FIGURE 4:**
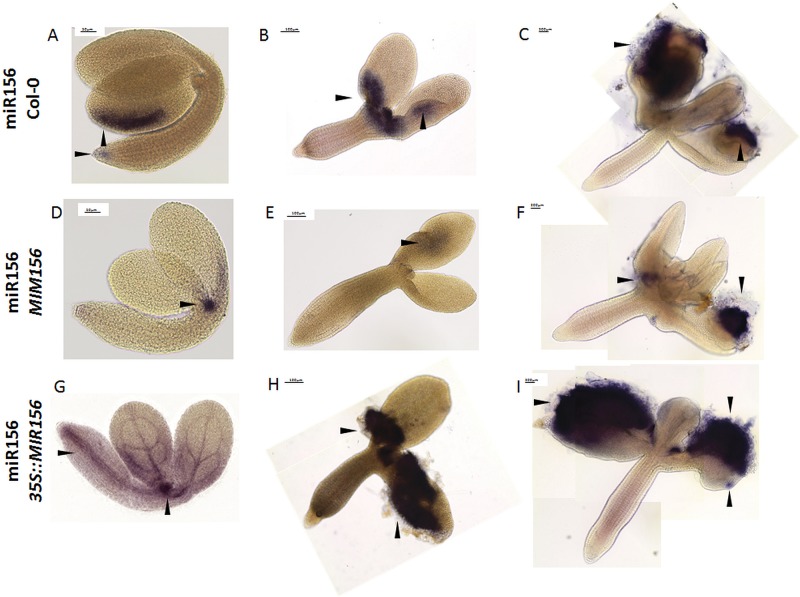
Whole mount *in situ* hybridization of the miR156 molecules in the explants undergoing SE induction in WT **(A–C)**, *MIM156* **(D–F)**, and *35S::MIR156* **(G–I)** at 0th day **(A,D,G)**, 5th day **(B,E,H)**, and 10th day **(C,F,I)**. Arrow heads indicate the detected signal.

The method was also tested to determine whether it could be used for the quantitative assessment of the analyzed miRNAs in the tissue during SE process. For this purpose, we attempted to detect miR156 during embryogenic induction in the *MIM156* line, which has a decreased level of miR156 molecules (Todesco et al., 2010; Cho et al., 2012) and in the *35S::MIR156* line, which over-accumulates miR156 (Wang et al., 2009; Jia et al., 2017). All of the samples were treated in parallel and the color reaction was performed in the same conditions. The intensity as well as the localization of the signal varied depending on the genotype and accordingly, a strong miR156 signal was observed in the SE-induced *35S::MIR156* explants, while the *MIM156* explants exhibited a much weaker WISH signal (**Figure 4**). The obtained results showed that the miRNA WISH procedure can be used to detect the differences in miRNA abundance, which was previously shown in the roots and zygotic embryos of Arabidopsis (Dastidar et al., 2016). It is worth mentioning that regardless of the intensity, the WISH signal of miR156 was localized in the cotyledons and SAM proximity of the SE-induced explants, i.e., in the tissue that is involved in SE induction (Kurczyńska et al., 2007). Hence, the role of miR156 in SE induction might be hypothesized given that the *SPL* (*SQUAMOSA PROMOTER BINDING PROTEIN LIKE*) genes have postulated to be among the candidate targets of miR156/157 in the SE of citrus (Wu et al., 2011), cotton (Yang et al., 2013), and Arabidopsis (Szyrajew et al., 2017).

**FIGURE 5:**
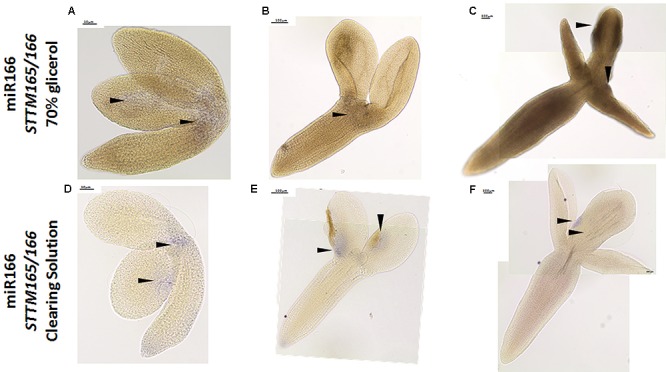
Whole mount *in situ* hybridization of the miR166 molecules in the explants undergoing SE induction in the *STTM165/166* line after treatment with 70% glycerol **(A–C)** and the clearing solution **(D–F)** at 0th day **(A,D)**, 5th day **(B,E)**, and 10th day **(C,F)** of culture. Arrow heads indicate the detected signal.

Additionally, we evaluated the usefulness of the WISH method for detecting miRNAs with a low abundance in the explants. To this end, we performed WISH for the miR166 in the *STTM165/166* line in which the miR165/166 molecules are degraded (Yan et al., 2012). Consistent with distinctly decreased accumulation of miR166 that was indicated in the RT-qPCR analysis (**Supplementary Figure S2**), the signal of WISH miR166 was barely visible in the SE-induced explants of *STTM165/166* (**Figures 5A–C**), in comparison to strong signal of miR166 during SE culture in WT (Wójcik et al., 2017). In order to improve the visibility of a WISH signal, we modified the standard procedure by replacing the treatment of the samples with 70% glycerol with a clearing solution (Bleckmann and Dresselhaus, 2016). We found that the resolution of the miR166 WISH signal was significantly improved in the cleared samples (**Figures 5D–F**). In conclusion, the use of the clearing solution instead of 70% glycerol might be recommended in order to increase the detection of the low-abundant RNAs, in particular, when relatively thick samples of *in vitro* cultured tissue are being analyzed.

**FIGURE 6:**
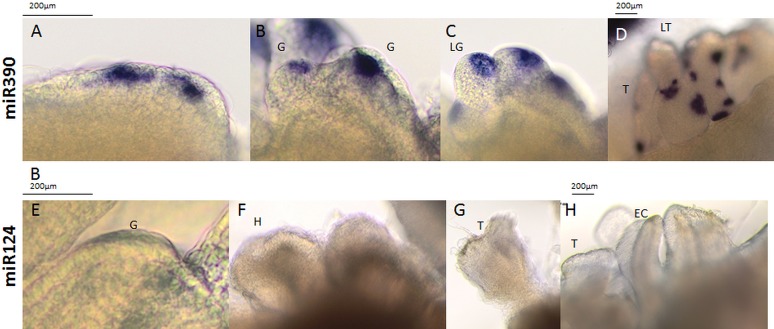
Whole mount *in situ* hybridization of the miR390 molecules **(A–D)** in the explants undergoing SE induction in WT on the 15th day of the culture. Probes against the mouse miR124 were used as the negative control **(E–H)**. All of the images show the somatic embryos during the globular (G), late globular (LG), heart (H), torpedo (T), late torpedo (LT), early cotyledonary (EC), cotyledonary (C), and mature stages of development (M).

The development of somatic embryos is often overlooked and significantly more attention is paid to the induction phase of SE. Several studies have shown a similarity in the somatic and zygotic embryos in terms of their morphological, histological, biochemical, and physiological aspects, and have introduced SE as a model for studying ZE (Zimmerman, 1993; Palada-Nicolau and Hausman, 2001; review in Winkelmann, 2016). However, there is a very limited number of publications that refer to the development of somatic embryos that is caused by the specificity of this material as well as limitations in the available analytical methods (Santos et al., 2006; Kumar et al., 2007; Kurczyńska et al., 2007; Nowak et al., 2012; Baskaran and Van Staden, 2017) and none of them has shown the molecular aspect of somatic embryo development. Performing WISH in explants that were undergoing SE in Arabidopsis enabled specific miR390 signals to be detected in the somatic embryos during different stages of development (**Figure 6**). A strong miR390 signal was detected in the subprotodermal explant tissue from which the somatic embryos might be formed (**Figure 6A**) as was also found by a cytological analysis (Kurczyńska et al., 2007). In line with a SAM-related expression of miR390 in maize (Douglas et al., 2010) and soybean (Wong et al., 2011), a WISH miR390 signal was also detected in tissue that corresponded with the SAM area of developing somatic embryos (**Figures 6B,C**).

Interestingly, somatic embryos that were at the torpedo stage of development displayed numerous isolated and intense miR390 signals that were located in the surface layer of the somatic embryo tissue (**Figure 6D**). Given that miR390 controls the lateral root development (Yoon et al., 2014) that has distinct molecular similarities with SE induction (Fehér et al., 2016), we assumed that the miR390 signals that were detected in the advanced SE culture may correspond to the formation of the secondary somatic embryos. The generation of secondary embryos by the primary somatic embryos is common in different plants (Santos et al., 2006; Priyono et al., 2010), including the embryogenic culture of Arabidopsis (**Supplementary Figure S3**).

Collectively, the miR390 signals that were observed in the somatic embryos of Arabidopsis (present results) together with the accumulation of miR390 in the somatic embryos of different species, including longan, (Lin and Lai, 2013), citrus (Wu et al., 2011), and larch (Zhang et al., 2012) suggest that miR390 might be a conserved regulator of somatic embryo development in plants.

**FIGURE 7:**
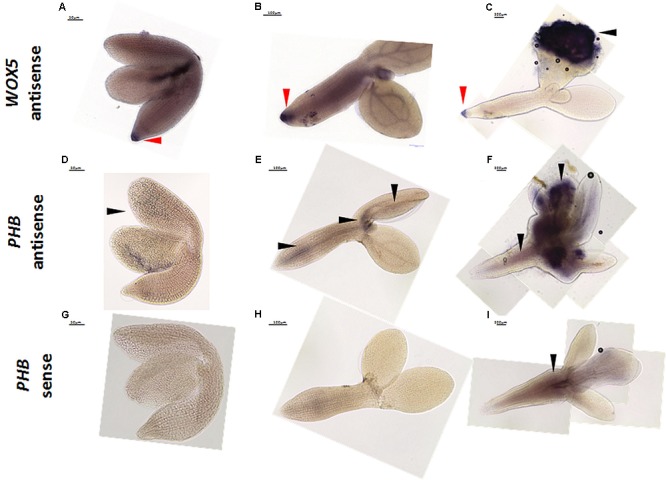
Whole mount *in situ* hybridization of *WOX5* **(A–C)** and *PHB* **(D–I)** in the explants undergoing SE induction at 0th day **(A,D,G)**, 5th day **(B,E,H)**, and 10th day **(C,F,I)**. The sense probes *PHB* **(G–I)** and *WOX5* (not shown) were used as the controls. Arrow heads indicate the detected signal; red arrow heads indicate the detected signal in RAM.

In the mRNA WISH as the positive control, we analyzed *WOX5* (*WUSCHEL-RELATED HOMEOBOX5*), which is expressed in the quiescent center in embryos and mature roots (Sarkar et al., 2007; Drisch and Stahl, 2015; Yan et al., 2016). The WOX5 signal for the antisense probe was detected in RAM at all of the analyzed time points (0, 5, 10 days) of the SE culture (**Figures 7A–C**). Consistent with the observation of Su et al. (2015a) that the *WOX5* expression preceded the development of somatic embryos by the embryogenic tissue, we detected an intense WOX5 signal in the SE-involved cotyledons that were induced for 10 days (**Figure 7C**). Then, the WISH for *PHABULOSA* (*PHB*), which is a target gene for the miR165/166 molecules (Tang et al., 2003; Vashisht and Nodine, 2014; Wójcik et al., 2017), was performed with antisense (**Figures 7D–F**) and sense (**Figures 7G–I**) probes. The signal of the antisense PHB probe was detected in the cotyledons of 0 d explants (**Figure 7D**) and in the proximity of SAM and vessels in the hypocotyl of 5 days explants (**Figure 7E**). In a more advanced SE culture (10 days), an intense PHB signal was found in the SE-involved upper parts of the explant including in the proximity of SAM and cotyledons (**Figure 7F**). However, we assumed that the hypocotyl-localized WISH signal might be a false positive background, as it was also detected by the PHB sense probe (**Figure 7I**). The spatiotemporal pattern of the *PHB* transcripts corresponded to the one that is observed during ZE in Arabidopsis (Gillmor et al., 2010) and is also similar to the localization of the PHB-GFP protein fusion during SE in Arabidopsis (Wójcik et al., 2017).

## Conclusion

**FIGURE 8:**
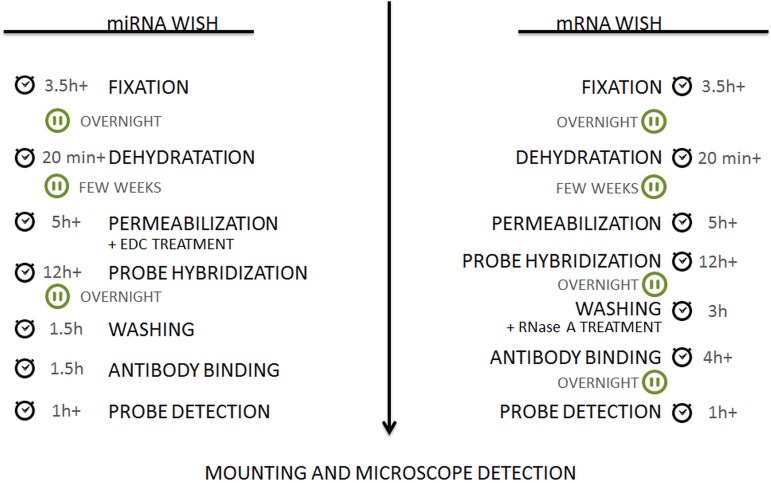
Flow chart of the protocols. The detailed procedure can be found in the section “Material and Methods.”

The protocols that are presented here, which enable the spatio-temporal localization of miRNAs and mRNAs in *in vitro* cultured tissue (**Figure 8**) are sensitive, efficient, and time saving as they do not require the long, time-consuming steps of preparing and sectioning tissues before hybridization. Thus, the recommended protocol enables the WISH signal to be monitored at different stages of a SE culture including the early and more advanced stages of SE in which the somatic embryos develop. The high sensitivity and specificity of the implemented miRNA-WISH with DIG-labeled LNA probes was proven by the very weak/absence of signal when using either the sense probes, animal miRNA (miR124) or when performing hybridization in the transgenic lines that had a reduced miRNA abundance.

## Author Contributions

AW, MG, and MN conceived and designed the research. AW and MM optimized the protocols. AW conducted the experiments. AW, MM, MN, and JK contributed to the imaging using an Olympus BX-43 microscope, Pannoramic FLASH 250 II scanning microscope, reagents, and SEM, respectively. AW and MG analyzed the data and wrote the manuscript. All authors read and approved the manuscript.

## Conflict of Interest Statement

The authors declare that the research was conducted in the absence of any commercial or financial relationships that could be construed as a potential conflict of interest.

## Supplementary Material

FIGURE S1 Relative amount of mature miR167 molecules at 0th, 5th, and 10th days of the Col-0 SE culture. Bars represent the standard deviation (*n* = 3). The relative transcript level was normalized to the internal control (*At4g27090*). Values that were significantly different from the 0 day are indicated with an asterisk (*P* < 0.05; *n* = 3 ± SD).

FIGURE S2 Relative amount of mature miR166 molecules at the 0th, 5th, and 10th days of the *STTM165/166* SE culture. Bars represent the standard deviation (*n* = 3). The relative transcript level was normalized to the internal control (*At4g27090*). Values that were significantly different from the Col-0-derived culture of the same age are indicated with an asterisk (*P* < 0.05; *n* = 3 ± SD).

FIGURE S3 Light microscopy and scanning electron microscopy images of the primary and secondary somatic embryos in the SE culture of Arabidopsis on the medium with 5 μM of 2,4-D. PSE – primary somatic embryos; SSE – secondary somatic embryos.
