# Supplementary figures and images for: Whole Mount in situ Localization of miRNAs and mRNAs During Somatic Embryogenesis in Arabidopsis
Source: Front Plant Sci. 2018 Sep 4;9:1277. doi: 10.3389/fpls.2018.01277 (PMC6131960; doi:10.3389/fpls.2018.01277)

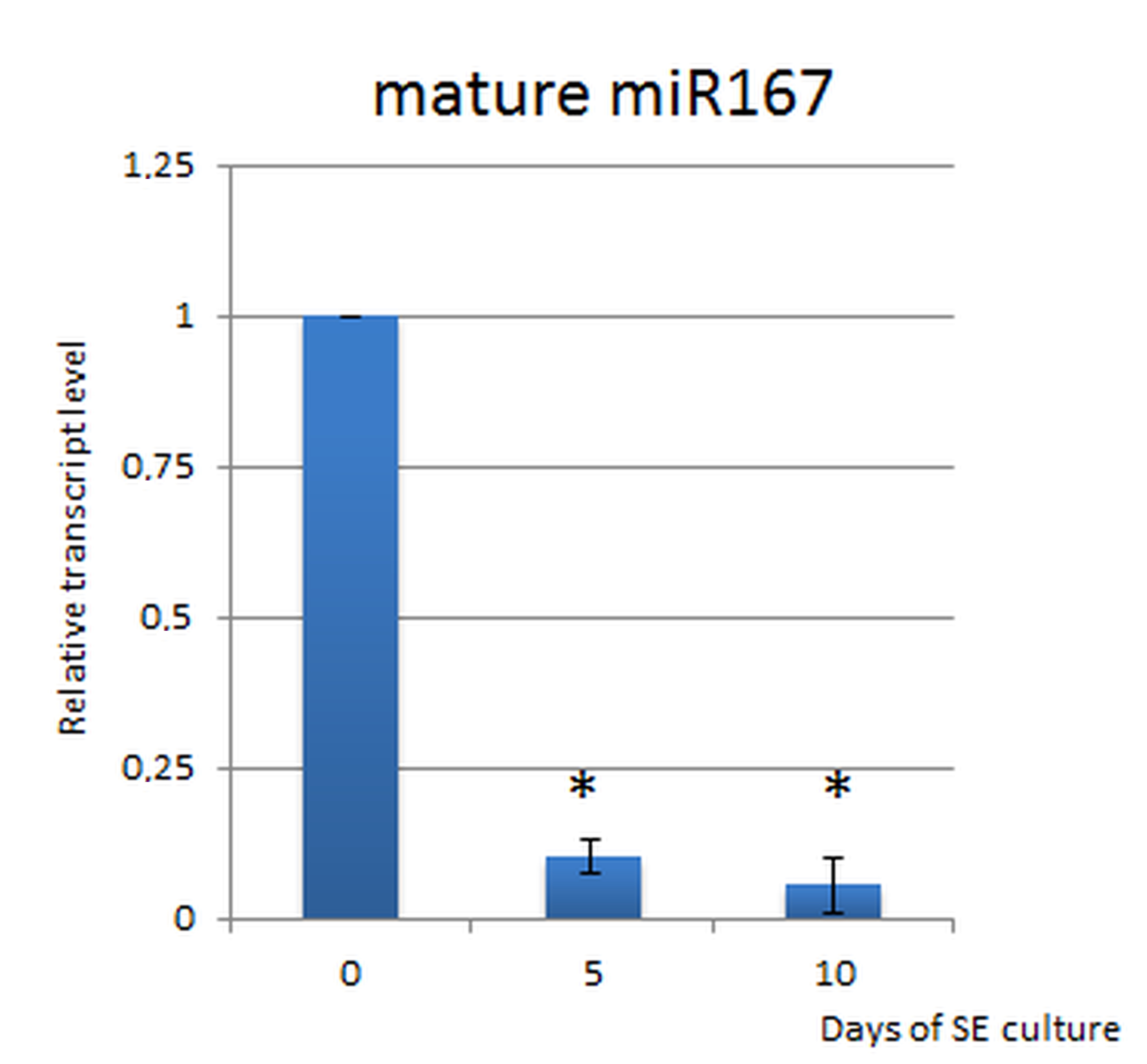

Supplement: FIGURE S1 — Relative amount of mature miR167 molecules at 0th, 5th, and 10th days of the Col-0 SE culture. Bars represent the standard deviation (n = 3). The relative transcript level was normalized to the internal control (At4g27090). Values that were significantly different from the 0 day are indicated with an asterisk (P < 0.05; n = 3 ± SD). [file Image_1.jpg]

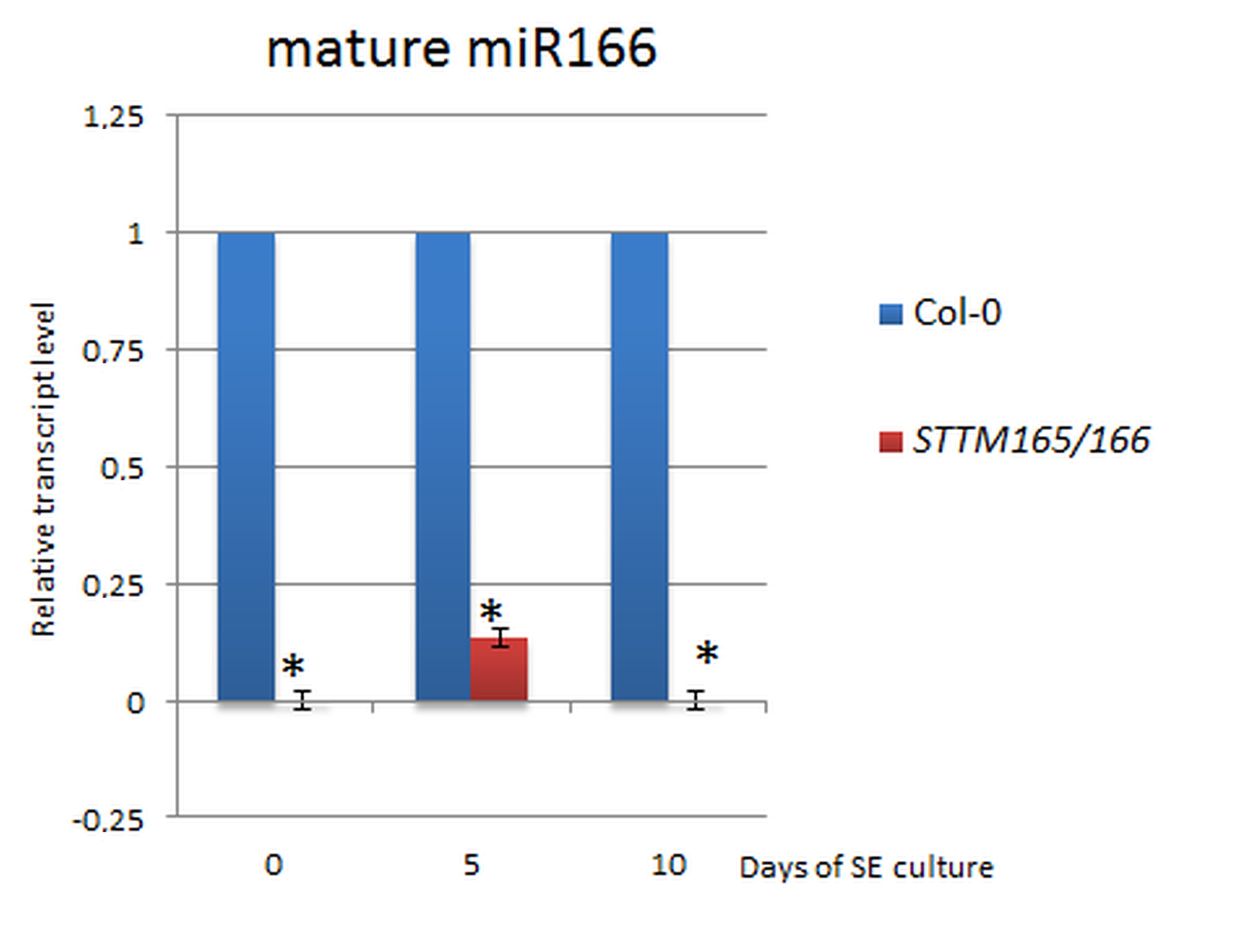

Supplement: FIGURE S2 — Relative amount of mature miR166 molecules at the 0th, 5th, and 10th days of the STTM165/166 SE culture. Bars represent the standard deviation (n = 3). The relative transcript level was normalized to the internal control (At4g27090). Values that were significantly different from the Col-0-derived culture of the same age are indicated with an asterisk (P < 0.05; n = 3 ± SD). [file Image_2.jpg]

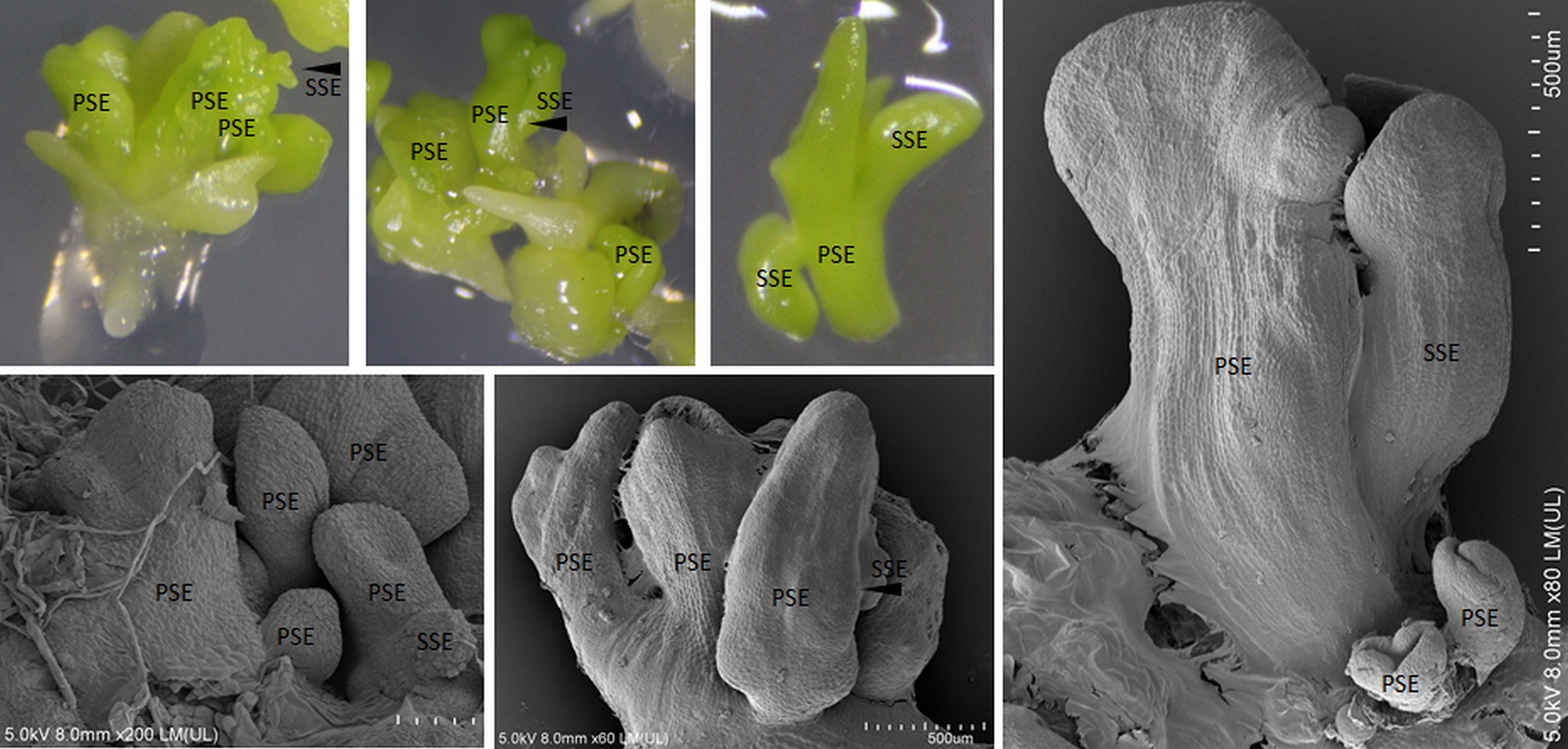

Supplement: FIGURE S3 — Light microscopy and scanning electron microscopy images of the primary and secondary somatic embryos in the SE culture of Arabidopsis on the medium with 5 μM of 2,4-D. PSE – primary somatic embryos; SSE – secondary somatic embryos. [file Image_3.JPEG]
